# Public Health Dashboards in Overdose Prevention: The Rhode Island Approach to Public Health Data Literacy, Partnerships, and Action

**DOI:** 10.2196/51671

**Published:** 2024-02-12

**Authors:** Jesse Yedinak, Maxwell S Krieger, Raynald Joseph, Stacey Levin, Sarah Edwards, Dennis A Bailer, Jonathan Goyer, Colleen Daley Ndoye, Cathy Schultz, Jennifer Koziol, Rachael Elmaleh, Benjamin D Hallowell, Todd Hampson, Ellen Duong, Abdullah Shihipar, William C Goedel, Brandon DL Marshall

**Affiliations:** 1 Department of Epidemiology Brown University School of Public Health Providence, RI United States; 2 AIDS Care Ocean State Providence, RI United States; 3 Parent Support Network Warwick, RI United States; 4 Rhode Island Department of Health Providence, RI United States; 5 Project Weber/RENEW Providence, RI United States; 6 Rhode Island College Providence, RI United States; 7 State of Rhode Island Executive Office of Health and Human Services Cranston, RI United States; 8 Center for Computation and Visualization Brown University Providence, RI United States

**Keywords:** community engagement, data dashboards, data literacy, health literacy, overdose, public health communication, public health surveillance

## Abstract

As the field of public health rises to the demands of real-time surveillance and rapid data-sharing needs in a postpandemic world, it is time to examine our approaches to the dissemination and accessibility of such data. Distinct challenges exist when working to develop a shared public health language and narratives based on data. It requires that we assess our understanding of public health data literacy, revisit our approach to communication and engagement, and continuously evaluate our impact and relevance. Key stakeholders and cocreators are critical to this process and include people with lived experience, community organizations, governmental partners, and research institutions. In this viewpoint paper, we offer an instructive approach to the tools we used, assessed, and adapted across 3 unique overdose data dashboard projects in Rhode Island, United States. We are calling this model the “Rhode Island Approach to Public Health Data Literacy, Partnerships, and Action.” This approach reflects the iterative lessons learned about the improvement of data dashboards through collaboration and strong partnerships across community members, state agencies, and an academic research team. We will highlight key tools and approaches that are accessible and engaging and allow developers and stakeholders to self-assess their goals for their data dashboards and evaluate engagement with these tools by their desired audiences and users.

## Introduction

Public health data dashboards are a critical communication tool for governments, health organizations, and academic research teams to bring rapidly emerging health surveillance information to a broader audience [1,2]. The rise of data dashboards in the United States during the COVID-19 pandemic resulted in a rapid proliferation of interactive maps, charts, and graphs and the wide dissemination of disease surveillance data by governmental organizations and research teams [3,4]. However, such data dashboards were in their infancy, and the governmental and research organizations creating them had urgent and multidimensional needs. Dashboards were expected to convey rapidly changing disease patterns, monitor emerging issues in local communities, and communicate about the risk of individual exposure [4,5]. Data dashboards are now a common platform for communicating policy changes and displaying trends for decision makers, researchers, and the news media [6]. Public health data dashboards created to monitor the overdose crisis and other ongoing public health issues across the United States are no different. Given their growing presence and appeal, the landscape of public health data dashboards leaves room for focused development and improvement in engaging users through accessible data storytelling, sustainable community partnerships, and data-driven decision-making [4,5,7-10]. The aim of this viewpoint paper is to describe the Rhode Island approach for data dashboard development, an instructional approach to overdose dashboard development driven by our experiences to date. First, we will illustrate our approach using 3 overdose data dashboard examples from Rhode Island; then, we will outline key considerations for dashboard data and accessibility, reciprocal partnerships, and pathways for action.

## Methods

### Public Health Data Literacy: PreventOverdoseRI

Rhode Island’s public data dashboard and educational information hub, PreventOverdoseRI [11] (nicknamed PORI), was launched in 2016 through a collaborative effort across state agencies, community organizations, and an academic research team. Making timely overdose surveillance data publicly accessible was critical to understanding the scale and scope of the growing overdose crisis and effectively distributing additional resources across the state during a period of rapidly rising fatal overdoses [9,12]. The first goal was to increase awareness of the overdose crisis by reaching a statewide public audience. Our next goal was to engage a subset of that audience as active dashboard users (ie, those who would stay on the website to interact with simple surveillance and resource maps and read educational infographics about overdose and the statewide policy response) [9,12].

**Figure 1:**
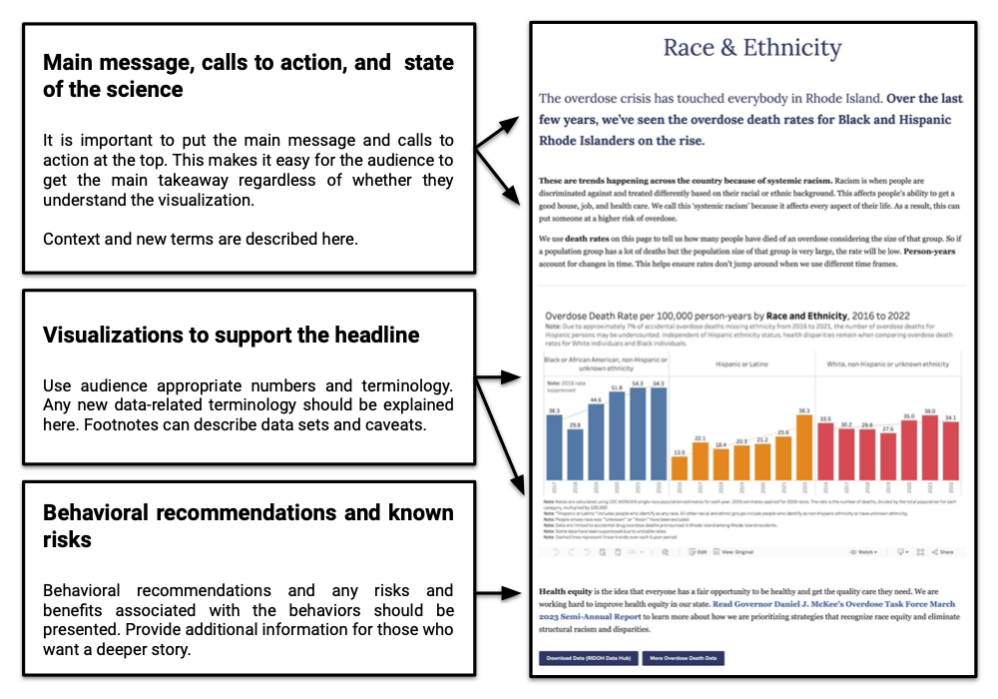
PreventOverdoseRI: wireframing and headlining content.

**Figure 2:**
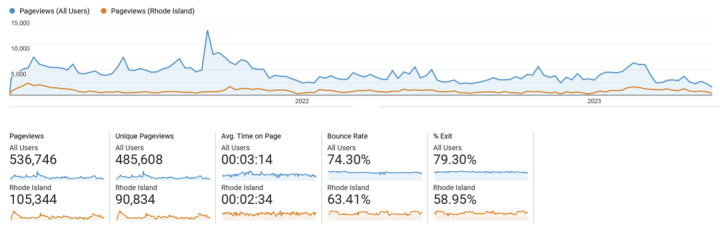
PreventOverdoseRI: evaluation metrics for public data dashboards.

Given the wide audience for this content, PORI was designed for accessibility and clear action steps by the users. This included descriptive headlines, simple data visualizations, and careful use of evidence-based tools such as the Center for Disease Control and Prevention (CDC) Clear Communication Index to assess content (Figure 1) [13]. Additional assessments included user testing of educational content and stakeholder involvement in data collection and dashboard dissemination [9,14]. Overdose surveillance data are visualized on PORI using the Tableau server [15]. Dashboard and educational content for PORI is hosted on a WordPress 6.3 website, using Google Analytics 4 (Google LLC) to monitor web traffic [16,17]. Evaluation of this type of data dashboard includes measuring levels of daily or monthly users and page views; duration of time and engagement on priority pages; and user pathway testing for understanding, ease of navigation, and follow-through to action, as demonstrated in Figure 2 [14]. Content development and improvement are ongoing, and user engagement with PORI continues to grow each year (with over 13,000 visits a month from 2018 to 2020 and 17,000 visits a month from 2020 to 2022).

### Meaningful Partnerships: The Vulnerability Investigation of Underlying Local Risk and Geographic Events Project

**Figure 3:**
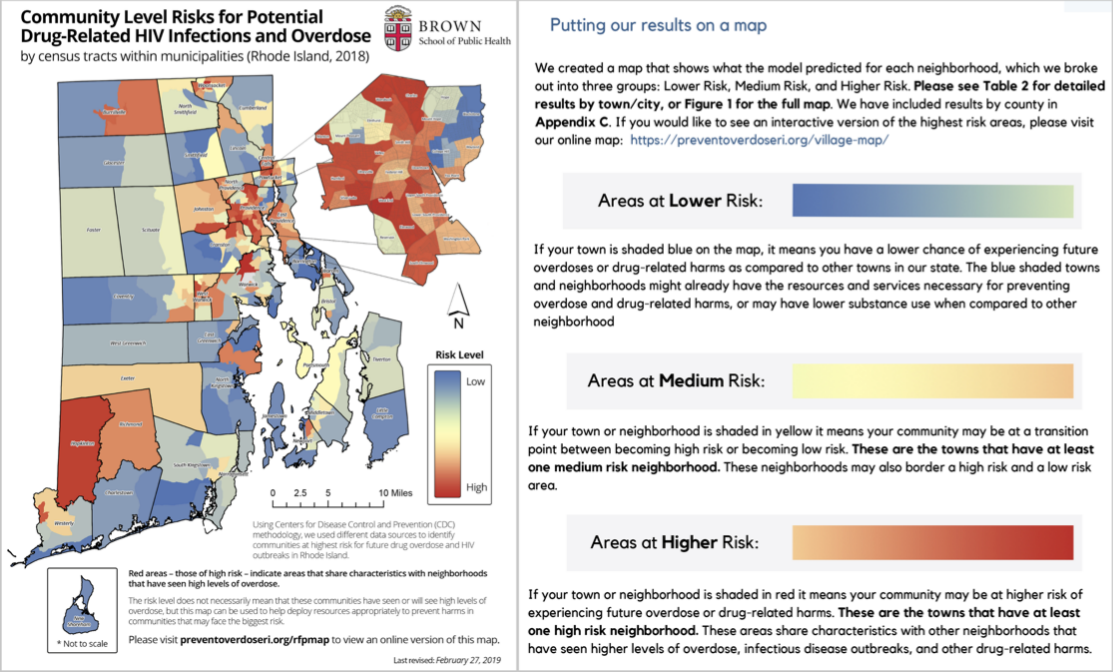
The Vulnerability Investigation of Underlying Local Risk and Geographic Events Project: contextualizing relative risk levels across the state.

In 2018, our academic research team conducted the Vulnerability Investigation of Underlying Local Risk and Geographic Events (VILLAGE) project, an evaluative public health project that aimed to identify areas at higher risk of increased drug-related harms relative to other areas of the state. For this type of data dashboard partnership, community organizations played a central role in conveying key messages and the dissemination of information to the informed public. The emphasis was on creating a simple, responsive data dashboard mapping tool for community organizations to inform harm reduction resource distribution at the community level. The goal was to create maps with census-tract level granularity to convey community and structural vulnerability to drug-related harms, such as overdose and HIV or hepatitis C outbreaks, as compared to other census tracts in the state [18,19]. Given the more specialized end users and emphasis on structural data and predictive modeling results visualized on a map, the data dashboard involved stronger user testing and evaluation of the maps from the Rhode Island Department of Health and community stakeholders. Our academic research team provided support for technical training on machine learning uses and worked with partners to draft language to convey the results of the predive model, named “vulnerability scores,” that prioritized census tracts for intervention. Community stakeholders helped lead the translation and dissemination of the VILLAGE project and accompanying materials (Figure 3) [18,19]. Results were visualized with ESRI ArcMap (version 10.4; Environmental Systems Research Institute, Inc) [20]. The final map and findings were printed for local distribution as well as hosted on the internet in PDF format. Evaluation of this work included the level of community engagement with dissemination activities, monitoring the relative allocation of resources as compared to vulnerability scores, and use of the census-tract-level vulnerability maps to secure additional grant funding.

### Pathways for Action: Preventing Overdose Using Information and Data From the Environment Community Randomized Trial

**Figure 4:**
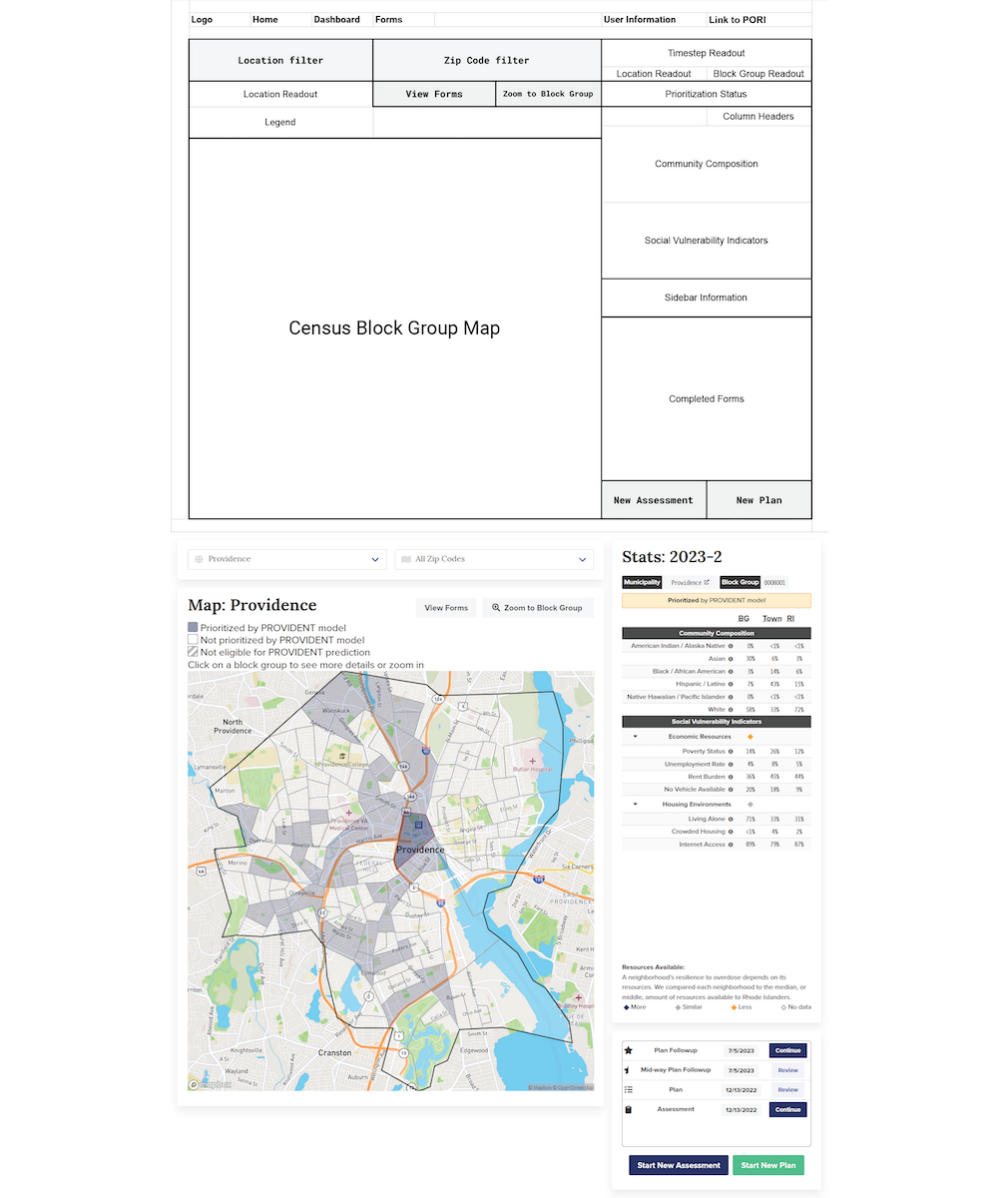
Preventing Overdose Using Information and Data From the Environment (PROVIDENT) community randomized trial: wireframing and mapping.

In 2021, our academic research team launched Preventing Overdose Using Information and Data From the Environment (PROVIDENT), a statewide, randomized, community-level trial designed to assess the impacts of proactive harm reduction resource allocation to census block groups prioritized based on a predictive analytics model [21]. While this data dashboard features increased complexity of available information for end users, the data dashboard is still organized for rapid usability and uptake, as illustrated in Figure 4. The PROVIDENT dashboard places a higher emphasis on tablet-friendly or field use of the dashboard maps, and the predictive model results are visualized by shading at-risk neighborhoods. The dashboard was developed in *Vue 3*, a JavaScript library, using Vega (University of Washington Interactive Data Lab) for visualizations, Google Firestore (Google LLC) for data storage, and Google Analytics 4 for monitoring web traffic and engagement [17,22-24]. Evaluation of the implementation work for this community trial includes structured focus groups by end users, pre- and posttest assessments of workshops and public health data literacy materials, and a better understanding of the multitude of reasons community stakeholders may engage with the data dashboard and how they use data to drive action.

## Discussion

### Building Context Using Positionality and Structural Data

A broad goal of public health data dashboards is to increase awareness of emerging issues and build trust through data sharing and transparency, while ultimately telling the “public health stories” of communities and connecting data to decision-making and results [1,25,26]. In communicating public health concerns, the organizations creating a data dashboard are in a powerful position to determine their audiences and users; define and narrate the issue for their selected groups; and make key decisions regarding what data are collected, how they are analyzed, and how they are visualized and interpreted [10,27]. The volume of data made available on the internet can vary widely, from a municipal or state “open data” portal that allows end users to view large collections of row-level data to the more traditional epidemiological surveillance report on a governmental website [26]. Critically, the decisions about which data to curate and include in a dashboard affect how risk factors and disease drivers are explained, whether structural determinants are identified or measured, and if disparate health outcomes are assessed or contextualized for the intended audience and presented in an accessible way [6,10,27,28].

Building trust is a prerequisite for any entity that wishes to develop a public health data dashboard. Data dashboards are now a crucial platform to scale up regional capacity for conveying critical public health messages and emerging changes, and they require continuous work to ensure audience engagement and dashboard usability [29]. As an academic research group, an important step in trust-building for these data dashboards was to name our positionality and connection to institutional resources in the partnership development process of each new dashboard. Positionality is a process used in community-engaged and qualitative research methods, where researchers and investigators explicitly name their affiliations, privileges, identities, and other relations to power structures as they engage in community partnerships and work to address gaps in resources across partnerships [30]. Examples of building trust and transparency include engaging local partners to help increase local information represented on the dashboard and improve the granularity of maps as part of that process (Table 1 contains additional examples). As representatives of research institutions, governmental organizations, or other structures of power, we must be careful not to repeat, minimize, ignore, or perpetuate long-standing harms of systemic, institutional, and personal racism against Black and other minoritized populations in the medical, research, governmental, and public health fields [31-35]. Rhode Island has a system of work groups sponsored by the Governor’s Overdose Prevention and Intervention Task Force (Task Force), including a racial equity work group whose work brings attention to disparate health outcomes in access to care and service delivery by highlighting gaps in data. Specifically, these stakeholders brought awareness to how underreported racial disparities in existing data sets, including methadone uptake and increases in death rates among Black and Hispanic Rhode Islanders, needed to be contextualized and displayed on PORI. Compounding the disparate health outcomes of the overdose crisis is the history of racialized criminalization of drug use, excessive use of policing, and overincarceration of Black and Hispanic or Latine Americans who use drugs [32,36,37]. Central to the work of addressing substance use and overdose through a public health data dashboard is the acknowledgment and identification of how racist structural and political forces create our current climate of health inequities and disparate health outcomes, drive our interpretation of data, and influence local action and resource allocation [36,37].

**Table 1:** Self-assessing public health data literacy and user engagement.

Design considerations	Self-assessment and tools
Capture the attention of your intended audience	Who is your intended audience? Bite, Snack, Meal: a tool to curate and edit content using the intended audience to adjust the level of detail. Headlining: the main headline clearly explains and interprets the main issue and key figures to engage your intended audience (Figure 1).
Making decisions about content to engage people	What is the story? Wireframing: organize information on the page so that the most impactful information is first, ending with the recommended action (Figures 1 and 4). Representation: community strengths, voice, and expertise are included and centered. Stories about overdose and drug use avoid stigmatizing terms and narratives.
Increase accessibility for intended users.	How is the story being told? Increase accessibility for intended users. Does the story use personal narrative, counts, percentages, or rates? What type of data storytelling resonates most with your audience? Use the CDC^a^ Clear Communication Index tool to assess health literacy and prioritization of health information (Figures 1 and 3) [13]. Make content accessible. Use readability tools (sixth- to eighth-grade US reading level). The Simple Measure of Gobbledygook was created for health information in English and has open-access web-based tools, or the Fleish-Kinkaid tool, which is included in common word processing applications [38,39].
Increase transparency with context and multiple types of data	Is the story the same across all groups and categories? How does our story change when looking at race, gender, geography, or incarceration experiences? What visualizations clearly illustrate these differences in health outcomes? Build transparency with detailed footnotes to clarify definitions, types of analyses, and limits of the data or metrics. Increase the likelihood of engagement with the data and content by choosing data that reflect the diversity of race, gender, age, and other key determinants of health.
Build user engagement with dashboard elements	Is there more to the story? Define structural racism and structural determinants of health and offer context about redlining or patterns of historical and structural investments or disinvestments. Create data that are interactive, and allow users to adjust timeframes, toggle between counts and rates, and map that zoom to zip code or census tract level if possible. Add local information, interactive maps, and relevant policy timelines to guide decisions and actions.
Create pathways for action	What does this mean for me? Make it personal for end users with a call to action on each page. Examples include connecting to additional education, encouraging harm reduction behavior change, offering referrals to care, or advocating for policy action.

^a^CDC: Centers for Disease Control and Prevention.

For PORI, where the goal is to engage a wide audience with public health data stories, the focus is on showing improved reporting on race and ethnicity across key data points related to health care engagement and outcomes during public Task Force meetings and visualizing both counts and rates on the dashboard whenever possible. Conversely, with the VILLAGE and PROVIDENT dashboards, the goal is to engage partners and create pathways for data-driven policy response and resource distribution. Therefore, those dashboards include additional neighborhood-level context, such as community demographics and social vulnerability indicators, which are chosen in collaboration with community stakeholders and can act as a proxy for identifying communities facing increased structural vulnerabilities (Figure 4).

In particular, creators of data dashboards have a responsibility to define, adjust, and disaggregate the types of data they collect from Black, Indigenous, Asian, Hispanic or Latin, and other marginalized racial and ethnic communities and contextualize why and how such data are collected and analyzed [27,28,35,40]. Reporting on health inequities and outcomes to a broad audience should include naming socially constructed categories of race, addressing the historical drivers of persistent and unequal health outcomes, and including more community-level reporting [28,40,41]. This is one such process that reveals how racism (rather than race) is the root cause of the differences we might observe by race and allows for disparities and inequities that may otherwise be hidden in the data to come forward. Ponce et al [27] speak to a wide range of data-driven approaches to improving data equity, which include disaggregating and contextualizing “racialized” identities in our US governmental data sets and the importance of visibility of additional ethnic identities as a means toward visibility and health outcomes. They draw out this concept further, citing the rapid growth of “multiracial” identities, and suggest other contextual questions to address the intersection of health and race: “depicting the health needs of multiracial people is more complex than a better measurement of group membership” [27].

Public health data dashboards also present the opportunity to increase the visibility of some structural systems of racism that create harm in the United States, particularly through the use of maps, data storytelling, and prioritization of resources in housing, education, health care, and transportation within a community. Overlays on maps highlight historical forces of segregation and structural disinvestment, such as through redlining maps, and reveal areas to be prioritized for resources and intervention [31,32,42]. Therefore, to begin addressing the structural harms and outcomes of systemic and institutionalized racism, data dashboard developers can create shared community power and oversight over definitions of race and other identifiers, the process of data collection, and increased use of data on structural drivers of health [40,43,44].

### Reciprocal Partnerships and Engaging Users

**Figure 5:**
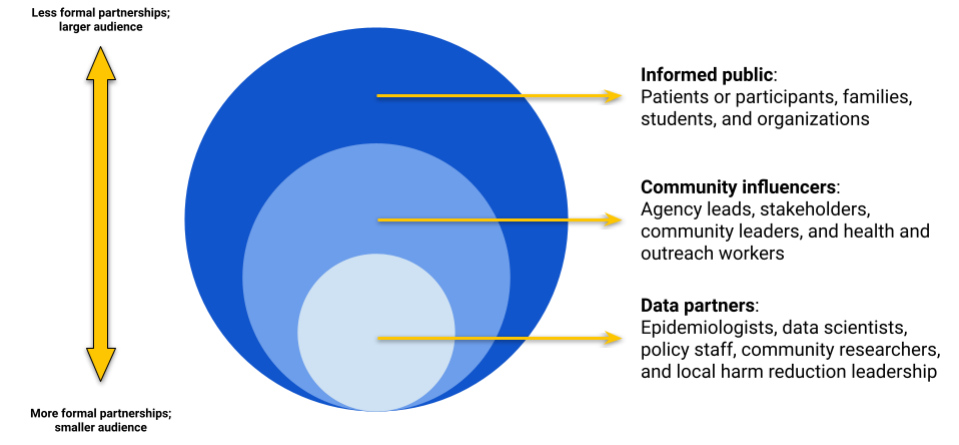
Establishing partnerships and engaging users.

The diverse components of a data dashboard have led to our curated approach to data sharing and intentional oversight by community stakeholders and users [9]. This system has been made possible by funding, collaboration, iteration, and strong community engagement and oversight from the extended overdose prevention and intervention community, including peer advocates, community organizations, governmental agencies, and our academic research center [9,14]. Assessing partnerships and defining and engaging an audience of potential dashboard users are among the first steps to creating a data dashboard, as outlined broadly in Figure 5. In Table 2, we detail 3 groups of decision makers who can share oversight for developing and creating a community-driven data dashboard and potential pathways for partnerships and cofunding. The broad decision-making groups include: (1) community stakeholders, including people with lived experience from a diversity of racial, ethnic, and cultural backgrounds; local nonprofits and leaders; and local policy makers closest to the issue who can intervene directly, cocreate, or improve data collection and interpretation, and advise on policy; (2) academic partners, or academic research teams, who have access to analytic and data security resources, partnership funding, national and international context, and methods for research and can develop evidence for interventions and policy change; and (3) governmental agencies who represent or own health and surveillance data, can help shape policy directives and resource distribution, and increase access to national funding.

**Table 2:** Defining data dashboard goals and partners.

Dashboard type	User goals	Decision-making partners
Public health data literacy (PORI^a^)	Dashboard users may interact with the headlines, engage in data visualizations such as maps and bar charts, review educational content, and use resource maps.	Community stakeholders advocate for their information needs and priorities. Give input on data governance, the interpretation of data, how to contextualize with mapping, and messaging to connect with the audience. Academic partners may host data dashboards, data visualization, and data-sharing security. Helps frame messaging and interpretation of data trends in the context of national or international trends. Provide analytic support and rigor. Governmental agencies coordinate volunteer working groups on data governance and support purchasing web resources. Helps frame messaging and interpretation of data trends from a policy leadership point of view. Determines analytic processes and partners. Secures funding.
Meaningful partnerships (the VILLAGE^b^)	Dashboard users interact heavily with the data visualizations and maps and offer expertise on education and resources.	Community stakeholders establish contracts with key community organizations, accountable to the government. Community organizations participate actively in the creation of data dashboards and are compensated for their time, meeting attendance, survey completion, and agreement to participate. Academic partners further develop data security measures, which may include protected log-ins for selective access. Data are readily available and contextualized, and headlines clearly interpret results and footnotes to help drive policy decisions and resources for intervention. Curated maps with increased detail are tested with community stakeholders. Governmental agencies establish contracts with key community organizations, are accountable to the government, and have resources to contribute. Funders may organize meetings with stakeholders to interpret or translate data collectively.
Pathways for action (PROVIDENT^c^)	Dashboard users influence how and what data are collected, analyzed, and visualized. They may request highly specific interactive features to improve the functionality and utility of the dashboard.	Community stakeholders: community organizations participate actively in the creation of data dashboards and are compensated for their time. Formal contracts and scopes of work are required to define each partner’s role. Academic partners may be a stronger leader in this role, providing dedicated research funding and engagement opportunities. Engaging community influencers by building shared language, training support for interpreting and contextualizing the data, and making data products that are highly actionable and usable for the community. Governmental agencies: governmental organizations engage with all partners to discuss the interpretation of data, refine data collection processes, and address missing information.

^a^PORI: PreventOverdoseRI.

^b^VILLAGE: Vulnerability Investigation of Underlying Local Risk and Geographic Events.

^c^PROVIDENT: Preventing Overdose Using Information and Data From the Environment.

The ways in which the decision-making groups interact and govern each project vary by dashboard type, as noted in Table 2. With PORI, the academic partners are highly responsive to the feedback and input of the community stakeholders as a means to increase the accessibility of the dashboard [9]. Content creation and data oversight from community stakeholders occur in public or semipublic meetings, which are often organized and facilitated by government agencies (ie, the Task Force and related working groups). For the VILLAGE project, community stakeholders advised on data set selection and helped create the dissemination plan, which included a community white paper, brief “1-pagers” to describe high-level results in lay terms, and a statewide conference with sessions created and led by the stakeholders [18,19]. Finally, for our PROVIDENT study, oversight of the dashboard is led by academic partners but governed through structured community advisory meetings and project team meetings with contracted community stakeholders and government agencies. Community stakeholders also help cofacilitate workshops and develop “1-pagers” as we scale up and disseminate the study results.

Next, in Figure 5, we define 3 types of dashboard partnerships to help guide editorial decision-making and content development, which should inform how data are displayed and contextualized for users and determine action steps. The first audience we focus on is the informed public, which includes the local audience that needs access to emerging public health headlines and visualizations that develop an overall data story or narrative. The second audience we identify is community influencers, key community organizations, and leaders in the public health response who have existing partnerships with governmental agencies. This audience can engage with maps and other interactive visualizations as active dashboard users and provide ongoing insights to help prioritize resources at the town or neighborhood level and identify structural disparities or emerging needs. Our last audience is the data partners, who include all stakeholders involved in cocreating and iterating on data dashboard tools, acting as users of the data to get ahead of emerging issues, address systemic disparities, highlight community strengths, and lead strategic planning and policy change.

### Operationalizing Public Health Data Literacy

We use a self-assessment process to think about engaging potential dashboard users with key headlines but also with interactive elements such as data visualizations and maps (Table 1). Some data elements work best when the intended user has some exposure to statistical concepts such as rates and percentages, geographical units, and an understanding of 1 or more public health data sets [45,46]. Data visualizations can quickly become complex and may use a number of analytic methods and software types that rely on an understanding of how to navigate and operate a user interface [47,48]. Best practices in user interface and data dashboard research suggest such products should be created in partnership with the intended users and monitored or revised continuously for improvement and engagement metrics, but resource constraints and hesitancy to use data dashboards for broader communication have limited the use of these tools in overdose prevention and intervention at the local level [4,7,45,47,49-52].

We identified a key set of skills to support wider audience engagement and accessibility, which we refer to as public health data literacy*.* Health literacy is defined in the United States as “when a society provides accurate health information and services that people can easily find, understand, and use to inform their decisions and actions” [53-56]. The concept and field of health literacy have been explored, researched, and further defined in the past 20 years to reveal differences in health outcomes based on individual “levels” of health and health systems literacy, highlighting the importance of accessible health communication and reliable information on accessing systems of care [56-58]. To contextualize this as it applies to public health dashboards, we suggest that numeracy, a component of health literacy, should be elevated as a core skill of data dashboard engagement. Numeracy is “the degree to which individuals have the capacity to access, process, interpret, communicate, and act on numerical, quantitative, graphical, biostatistical, and probabilistic health information needed to make effective health decisions” [46,59]. Establishing health literacy levels, including numeracy, can be achieved using evidence-based tools for assessing web-based content. We recommend reviewing individual dashboard pages using the CDC Clear Communication Index [13]. Designing a data dashboard with these skills in mind affects how the user engages with the dashboard and their ability to act on data-based recommendations, not just at the individual level with dashboards such as PORI, but at the organizational, community, and policy levels using data from forecasting-driven dashboards such as the VILLAGE and PROVIDENT. Therefore, we think of public health data literacy as how our users collect, interpret, and understand the public health data we need and use it to guide community-level action and policy decisions for better health outcomes and systemic change*.*

To increase trust in public health data dashboards, we recommend health literacy and accessibility efforts be done in tandem with community stakeholder partnerships for improved creation, interpretation, and prioritization of results and action [9,29,60-62]. As academic partners, we paired public health training sessions with ongoing feedback sessions. These interactive training sessions were first developed as an introduction to dashboard storytelling with PORI and soon evolved to include public health terminologies such as counts, percentages, rates, and relative risk for the VILLAGE project. Ultimately, as the dashboard complexity grew, we created a workshop for the PROVIDENT study to discuss the processes behind prediction and forecasting, as well as the other concepts from previous training sessions. In Table 1, we focus on our latest self-assessment questions to develop and contextualize the data and present it as a narrative or story, outlining key questions the story should answer for optimal user engagement and action. Each question in Table 1 is accompanied by a list of suggested tools for editing, curating, and contextualizing the data. We begin with the self-assessment question, “Who is the audience?” We suggest that most audiences benefit when information is curated to the key conclusions, written as headlines, to drive dashboard usage and engagement with the content. Here we use the marketing tool “Bite, Snack, Meal” to refer to how much of the data story to share with each audience [63]. For example, highly curated data and easy-to-remember headlines are useful for nearly all audiences to broadly understand and engage with the issue and are considered the “bite.” On the other hand, the deep dive into the data is the “meal” and should be reserved for the most engaged stakeholders and users. Next, we ask, “What is the story?” We offer specific suggestions for the organization and structure of the story on the data dashboard page for the greatest accessibility and understanding. The following 3 questions—“How is the story being told? Is the story the same across all groups and categories? Is there more to the story?”—each refers to dimensions of the issue and engagement to ensure sufficient context and connection with key issues and methodologies used. There are specific tools listed to address numeracy, health disparities, and historical or political context within each of those questions. Finally, “What does this mean for me?” is our data dashboard action step. Each data dashboard page can lead to the desired action steps for the user to go beyond the data, such as links to education, connections to local care and resources, or opportunities for community and policy engagement.

### Summary

The lessons learned across 3 overdose dashboard projects in Rhode Island offer insights into the complexity of providing data to engage communities and using data to drive action on emerging issues. PORI illustrated for us how research and governmental institutions that provide data have a significant role to play in making the data accessible and in addressing the complexity of disparate health outcomes and data equity in our communities. Data partners such as Rhode Island’s Executive Office of Health and Human Services lead with a framework for centering racial justice, stating openly, “We acknowledge and seek to address the historical and ongoing harm caused by systemic oppression, including the role of state and non-state actors in perpetuating these harms.” [64]. Furthermore, community engagement and partnerships are essential to building a reciprocal relationship, trust, and accountability with the dashboard users, as we explored in the VILLAGE project. As noted by Ponce and colleagues [27], “For a solid foundation, there must be visibility of marginalized communities throughout the pipeline of data equity, that is, from data collection or capture to data use to how data are used and by whom.” [27]. Finally, through the PROVIDENT community trial, we saw the powerful insights brought about through cocreated dashboards and stronger formalized and compensated partnerships. Similar conclusions were drawn from the HEALing Communities Study: “dashboards can be used to foster community-driven solutions to address the opioid overdose epidemic” [52].

### Conclusion

Public health and overdose surveillance and dashboarding methods will continue to evolve as new public health crises emerge and software and web tools enable broad dissemination. Given the scale and escalation of changes in the overdose crisis and other emerging public health issues, the need for rapid and effective communication and local response will likely remain rooted in the key concepts learned thus far: accessibility, partnerships, and action. The approach presented herein offers accessible and concise steps that local governments and academic research partners can take to proactively engage and cocreate advanced epidemiological content with communities while preparing for the increasing demand and rapid scale-up of public health data visualization and communication during times of highest need.

## Abbreviations

CDC: Centers for Disease Control and Prevention
PORI: PreventOverdoseRI
PROVIDENT: Preventing Overdose Using Information and Data From the Environment
Task Force: Governor’s Overdose Prevention and Intervention Task Force
VILLAGE: Vulnerability Investigation of Underlying Local Risk and Geographic Events
